# Phenotypic Diversity Analysis and Integrative Evaluation of *Camellia oleifera* Germplasm Resources in Ya’an, Sichuan Province

**DOI:** 10.3390/plants14142249

**Published:** 2025-07-21

**Authors:** Shiheng Zheng, Qingbo Kong, Hanrui Yan, Junjie Liu, Renke Tang, Lijun Zhou, Hongyu Yang, Xiaoyu Jiang, Shiling Feng, Chunbang Ding, Tao Chen

**Affiliations:** College of Life Science, Sichuan Agricultural University, Ya’an 625014, China; zsh4266@163.com (S.Z.); kqb666666@163.com (Q.K.); yan15198254383@163.com (H.Y.); 13350950617@163.com (J.L.); trk20001126@163.com (R.T.); zhoulijun@sicau.edu.cn (L.Z.); yhy4868135@163.com (H.Y.); jiangxyu1997@outlook.com (X.J.); fenshiling@outlook.com (S.F.)

**Keywords:** *Camellia oleifera*, Germplasm resources, Phenotypic diversity, DUS test guideline

## Abstract

As a unique woody oil crop in China, *Camellia oleifera* Abel. germplasm resources show significant genetic diversity in Ya’an City. This study measured 60 phenotypic traits (32 quantitative, 28 qualitative) of 302 accessions to analyze phenotypic variation, establish a classification system, and screen high-yield, high-oil germplasms. The phenotypic diversity index for fruit (H’ = 1.36–1.44) was significantly higher than for leaf (H’ = 1.31) and flower (H’ < 1), indicating genetic diversity concentrated in reproductive traits, suggesting potential genetic variability in these traits. Fruit quantitative traits (e.g., single fruit weight CV = 35.37%, fresh seed weight CV = 38.93%) showed high genetic dispersion. Principal component analysis confirmed the fruit factor and economic factor as main phenotypic differentiation drivers. Quantitative traits were classified morphologically, and correlation analysis integrated them into 13 key indicators classified using LSD and range methods. Finally, TOPSIS evaluation selected 10 excellent germplasms like TQ122 and TQ49, with fruit weight, fresh seed yield, and kernel oil content significantly exceeding the population average. This study provides data for *C. oleifera* DUS test guidelines and proposes a multi-trait breeding strategy, supporting high-yield variety selection and germplasm resource protection.

## 1. Introduction

*Camellia oleifera* is a evergreen small tree or shrub in the genus *Camellia* of the Theaceae family, with leaf blade leathery and varied in shape, concavity or even vein, leaf margin serrate, leaf tips and bases vary in form; white or pink flowers, rich petal morphology; capsules, spherical or ovoid and predominantly green or yellow-green in color, rich diversity of fruit states; Seeds are predominantly renal and vary in color [1]. and is one of the endemic woody oilseed crops widely distributed in the southern region of China [2]. The oil content of *C. oleifera* seeds is 40–60%, and the extracted Camellia oil is rich in oleic acid, linoleic acid and other active ingredients with both nutritional functions and commercial development potential, which is a nutritious high-quality edible oil, and has been praised as “Eastern Olive Oil” by the Food and Agriculture Organization of the United Nations (FAO) [3]. Meanwhile, *C. oleifera* is also an excellent industrial raw material, widely used in soap, cosmetics, rubber and other industries [4]. The development of *C. oleifera* industry is of great strategic significance to guarantee national food and oil security, promote the economic development of mountainous areas and rural revitalization [5]. *C. oleifera* showed significant genetic diversity in leaf, flower and fruit morphology, and had excellent traits such as cold hardiness, drought resistance, pest resistance, high yield and high oil yield among its germplasm resources, showing outstanding genetic improvement potential [6]. In order to systematically protect and utilise the germplasm resources of *C. oleifera* the National Forestry Administration of China has issued the Technical regulations for the investigation and catalogue of genetic resources on *Camellia* spp. [7].

Ya’an City is located in southwest China, the transition zone between the Sichuan Basin and the Qinghai-Xizang Plateau, with a subtropical humid monsoon climate, an area of 94%, an elevation gradient from 500 to 5000 m, significant topographic heterogeneity, and a unique combination of water and heat and a vertical belt spectrum that has nurtured rich and unique *C. oleifera* germplasm resources, which is an ideal area for studying the phenotypic diversity of *C. oleifera* [8]. Phenotypic diversity analysis is the cornerstone of *C. oleifera* germplasm resource research by quantifying the morphological variation of organs and the degree of genetic dispersion of the population, which provides the basis for phenotypic anchoring for the analysis of the genetic structure of *C. oleifera* [9,10]. Qualitative traits are controlled by single or oligo genes, the inheritance pattern follows Mendel’s law, and the phenotypes show typical non-continuous distribution, which is characterized by significant genetic effect and precise phenotypic discrimination [11], and it is the core entry point for the analysis of the genetic mechanism of *C. oleifera* and the targeted improvement of *C. oleifera* varieties; Quantitative traits are regulated by multiple gene networks and characterized by continuous distribution of population phenotypes and environmental sensitivity [12], which are key targets for genetic improvement of *C. oleifera*, among which fruit quality and seed kernel oil content are the core indexes for evaluation of *C. oleifera* germplasm resources and breeding selection [13]. Based on the International Union for the Protection of New Varieties standard [14], the characterization of qualitative and quantitative traits of *C. oleifera* flowers, leaves, fruits and branches in terms of specificity, uniformity and stability (DUS) is the basis for building a new variety protection system. However, the current DUS test guideline under the UPOV framework is designed for broadly defined oleaginous Oil-tea *Camellia* plants [15], which does not adequately consider *C. oleifera* specific traits, and quantitative trait grading standards are missing, resulting in its inability to be accurately applied to the specific characterization of *C. oleifera* phenotypes. Therefore, integrating the multidimensional phenotypic comprehensive evaluation system of qualitative-quantitative traits is an inevitable way to improve the defects of the existing DUS test guidelines and realise the efficient utilization of *C. oleifera* germplasm resources [16,17,18].

The objectives of this study were (i) to explore the variation characteristics and diversity of the phenotypic traits of *C. oleifera* germplasm resources in Ya’an City, Sichuan Province; (ii) establish a framework for grading the phenotypic traits such as flowers, leaves, and fruits, which will serve as a reference for the establishment of a test for specificity, consistency, and stability of *C. oleifera*; (iii) To conduct a comprehensive analysis of economic traits, and screen out *C. oleifera* germplasm resources with excellent comprehensive performance, so as to provide a reference for the selection and breeding of *C. oleifera* varieties. This study will help to promote the establishment of DUS test guidelines for *C. oleifera* and better comprehensive utilization of *C. oleifera* germplasm resources.

## 2. Results

### 2.1. Diversity of Qualitative Traits

#### 2.1.1. Plant and Branch Phenotypes

**Figure 1:**
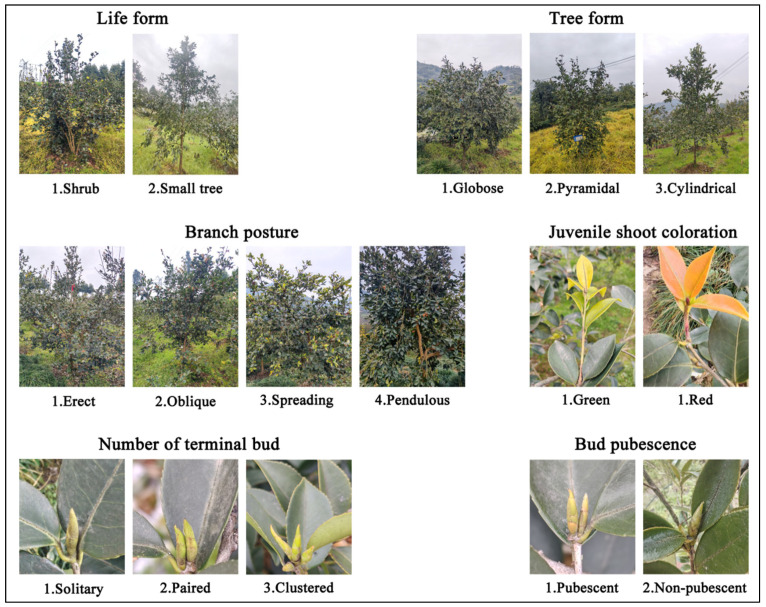
Grading standards for plant and branch qualitative traits of *Camellia oleifera*.

The life forms of *C. oleifera* germplasm resources in Ya’an City were dominated by shrub (45.70%) and small tree (54.30%) growth habits, with low phenotypic differentiation (H’ = 0.69) (Figure 1, Table 1). Tree form polymorphism was significant with pyramidal (56.29%) as the dominant phenotype followed by globose (34.11%) and cylindrical (9.60%). The branch phenotypes were significantly differentiated (H’ = 1.09), with oblique (54.64%) being the most predominant phenotype and pendulous (4.64%) being the rare phenotype; the color of the juvenile shoot coloration was bimodal in green (84.11%) and red (15.89%) (H’ = 0.44), with a low degree of genetic variability. The number of terminal buds was dominated by solitary (55.30%) and clusters (35.76%), and only 8.94% were paired; bud pubescence was pubescent (92.38%), and phenotypic conservatism was strong (H’ = 0.27).

#### 2.1.2. Leaf and Flower Phenotypes

**Figure 2:**
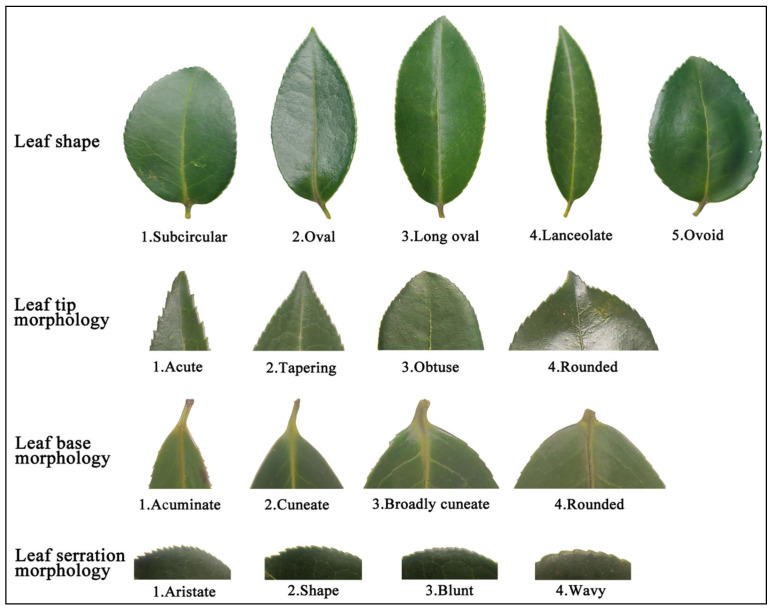
Grading standards for leaf qualitative traits of *C. oleifera*.

The leaf morphology of Ya’an *C. oleifera* germplasm resources was rich (Figure 2). Leaf shape was divided into 5 classes, the largest proportion was oval (52.32%), lanceolate was the smallest proportion of 7.62%, and the Shannon-Wiener index (H’) was 1.31, which was genetically rich (Table 1). 90.73% of the samples had convex veins and only 9.27% of the veins showed a concave pattern with a Shannon-Wiener index (H’) of 0.31. Leaf surface morphology was divided into 3 classes, with leaf surface flatness (58.28%) most and leaf surface revolute (3.97%) least. Leaf tip, leaf base and serration morphology were all categorized into four types, leaf tip was mainly tapering (48.34%), rounded (4.64%) was more rare in the samples, while leaf base was mainly cuneate (50.99%), and the type of serration morphology was more balanced, with Shannon-Wiener indices (H’) of 1.17, 1.21, and 1.10 for the leaf tip, base and serration morphology, respectively, which were all genetically more diversified.

**Figure 3:**
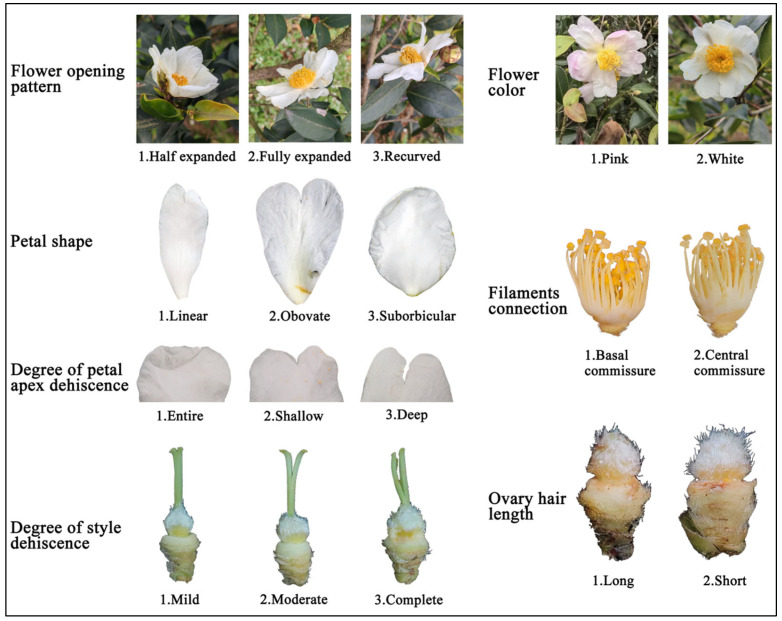
Grading standards for fiower qualitative traits of *C. oleifera*.

Flower morphology is more distinctive (Figure 3). Among the 8 traits of flowers, flower opening morphology, petal shape, degree of petal apex dehiscence, petal surface morphology and degree of style dehiscence were categorized into three classes, and filament connection, flower color and length of ovary hairs were categorized into 2 classes (Table 1). Flower opening morphology was mainly full exhibition (81.13%), petal shape was linear (26.49%), obovate (50.00%) and suborbicular (23.51%), the degree of petal apex dehiscence was mainly entire (89.07%), the petal surface morphology were mainly in wavy (50.99%), and the style dehiscence was mainly lobed (56.29%). The percentage of white flowers was 99.01%, and only 0.99% of flowers were pink, with a highly conservative genetic expression, highly similar to the *C. oleifera* germplasm resources in Hainan Province [19]. The filament connections mainly showed basal connivance (62.25%), and most of the samples (70.20%) had short ovary hairs. The Shannon-Wiener indices (H’) of the 8 traits were lower than 1 except for petal shape (H’ = 1.04), and Ya’an *C. oleifera* germplasm resources were genetically more conservative in terms of flower morphology.

#### 2.1.3. Fruit Phenotypes

**Figure 4:**
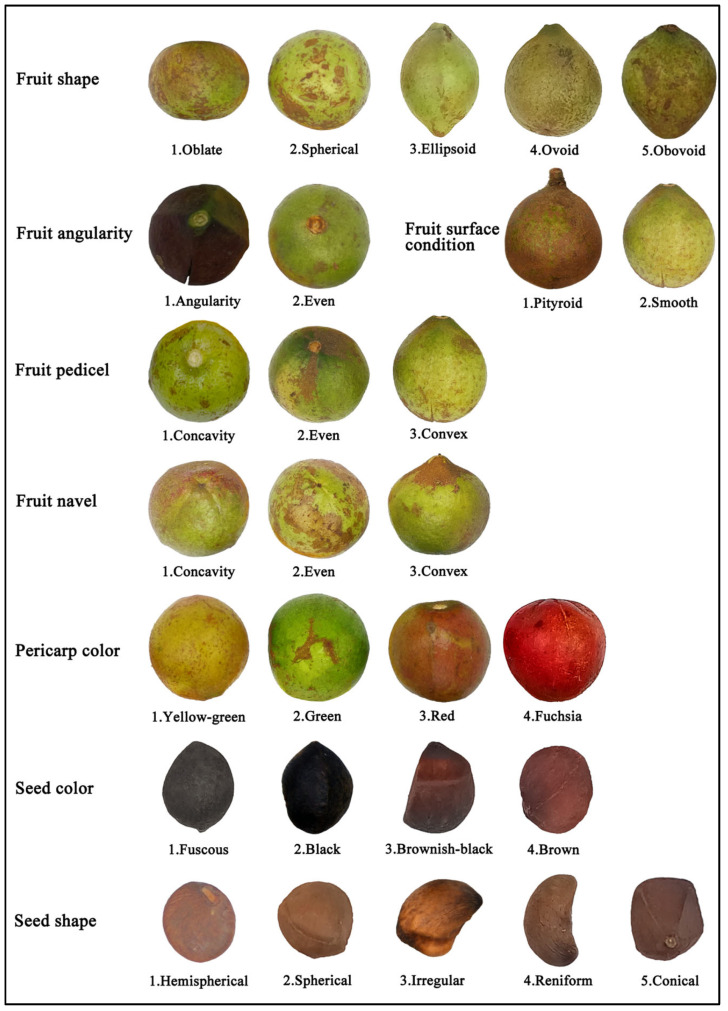
Grading standards for fruit phenotypic traits of *C. oleifera*.

Fruit phenotypes of *C. oleifera* germplasm resources in Ya’an City show high diversity characteristics (Figure 4). Both fruit shape and seed shape were categorized into 5 classes. Fruit traits were dominated by spherical (47.68%) as dominant phenotype, obovoid fruit (4.64%) as rare phenotype, oblate (13.91%) and ellipsoidal (12.25%) were distributed in equal proportions, and ovoid accounted for 21.52% of the fruit; seed shape was dominated by reniform (40.07%), spherical (4.97%) was a rare phenotype, hemispherical (20.20%) and irregular (20.53%) were distributed in equal proportions, and conical accounted for 14.24%, with Shannon-Wiener indices (H’)of 1.36 and 1.44, respectively, with rich genetic diversity. The pericarp color covered 4 types, with green (59.27%) and yellowish-green (33.77%) dominating, and red (6.62%) and fuchsia (0.33%) being rare phenotypes; the seed color was uniformly polymorphic, with fuscous (31.46%) and brownish-black (37.09%) accounting for a larger proportion, brown accounting for the smallest proportion (9.93%) and black (21.52%). Among the remaining fruit traits, the proportion of ribs was balanced, umbilicus depression (44.37%) and tip flatness (38.74%) were in a complementary distribution, tip prominence (16.89%) was a low-frequency phenotype, and pityroid accounted for 77.18% of the total, which reflects the adaptive differentiation of micromorphological traits.

### 2.2. Diversity and Rating of Quantitative Traits

#### 2.2.1. Analysis of Variance and Normality Test

**Figure 5:**
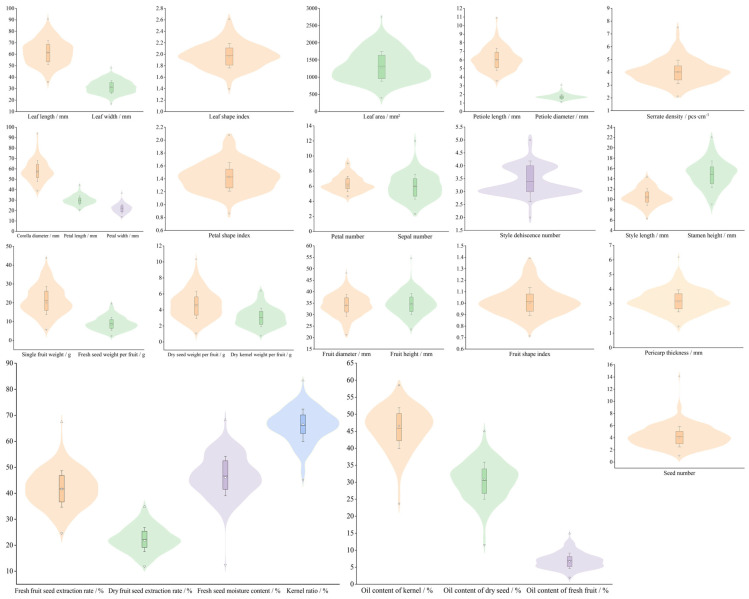
Violinogram distribution of 32 quantitative traits in 302 *C. oleifera* germplasm resources.

**Figure 6:**
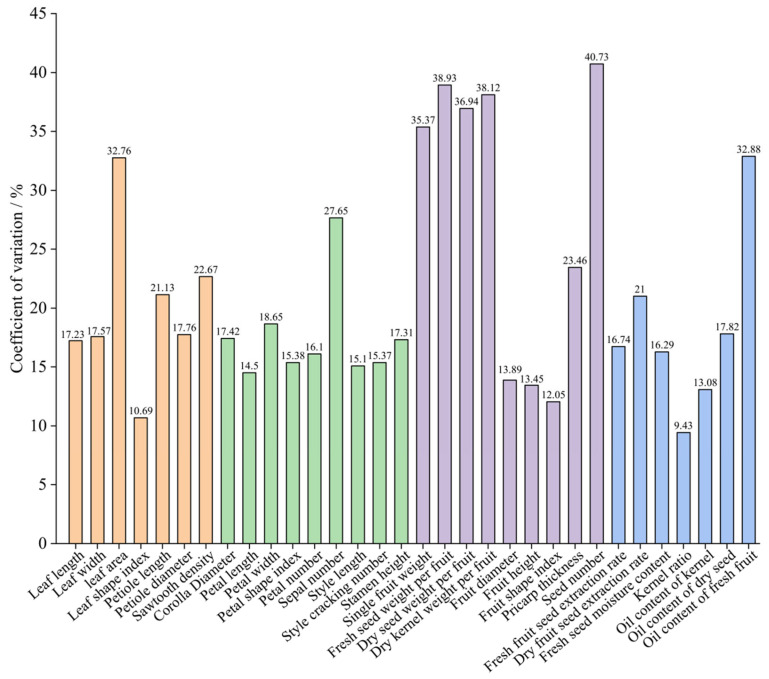
Coefficient of variation for 32 quantitative traits.

The 32 quantitative traits of 302 *C. oleifera* germplasm resources in Ya’an were symmetrically distributed (mean ≈ median) (Figure 5), supporting the representativeness of the sample population. Among the leaf and flower traits, leaf area phenotypic plasticity was the highest (395.03–2750.92 mm^2^, CV = 32.76%) (Figure 6), followed by sepal number (2.33–12.00, CV = 27.65%) and sawtooth density (2.10–7.50 pcs/cm, CV = 22.67%), and coefficients of variation (CV) of the remaining traits ranged from 10.69% to 20.00%, indicating high overall genetic redundancy for leaf and flower traits. The leaf shape index was stable (1.39–2.61, CV = 10.69%), consistent with the previous study [20].

The phenotypic differentiation of fruit traits was significant with coefficient of variation (CV) ranging from 9.43% to 40.73% (Figure 6). The single fruit weight (5.73–43.88 g, CV = 35.37%), the fresh seed weight per fruit (2.28–19.56 g, CV = 38.93%) (Figure 5), the dry seed weight per fruit (1.04–10.36 g, CV = 36.94%), and the dry kernel weight per fruit (0.75–6.39 g, CV = 38.12%) all exhibited high genetic dispersion (CV > 35%), suggesting significant genetic differentiation in fruit and seed weight traits and providing a phenotypic foundation for the screening of high-yield germplasm. The average fruit shape index was 1.01 (spherical), and the pericarp thickness (1.46–6.19 mm, with an average of 3.20 mm) was significantly lower than that of the population in Shangcheng County [21]. The coefficient of variation of the number of seeds per single fruit was the largest (1.00–14.09, CV = 40.73%), which was in line with the trend of similar studies [22]. Among the economic traits, the stability of the kernel ratio was the highest (CV = 9.43%), while the variation of the oil content of fresh fruits was significant (CV = 32.88%), confirming the gene-environment interaction effect in the lipid metabolism pathway of *C. oleifera* [23].

#### 2.2.2. Normality Test

**Figure 7:**
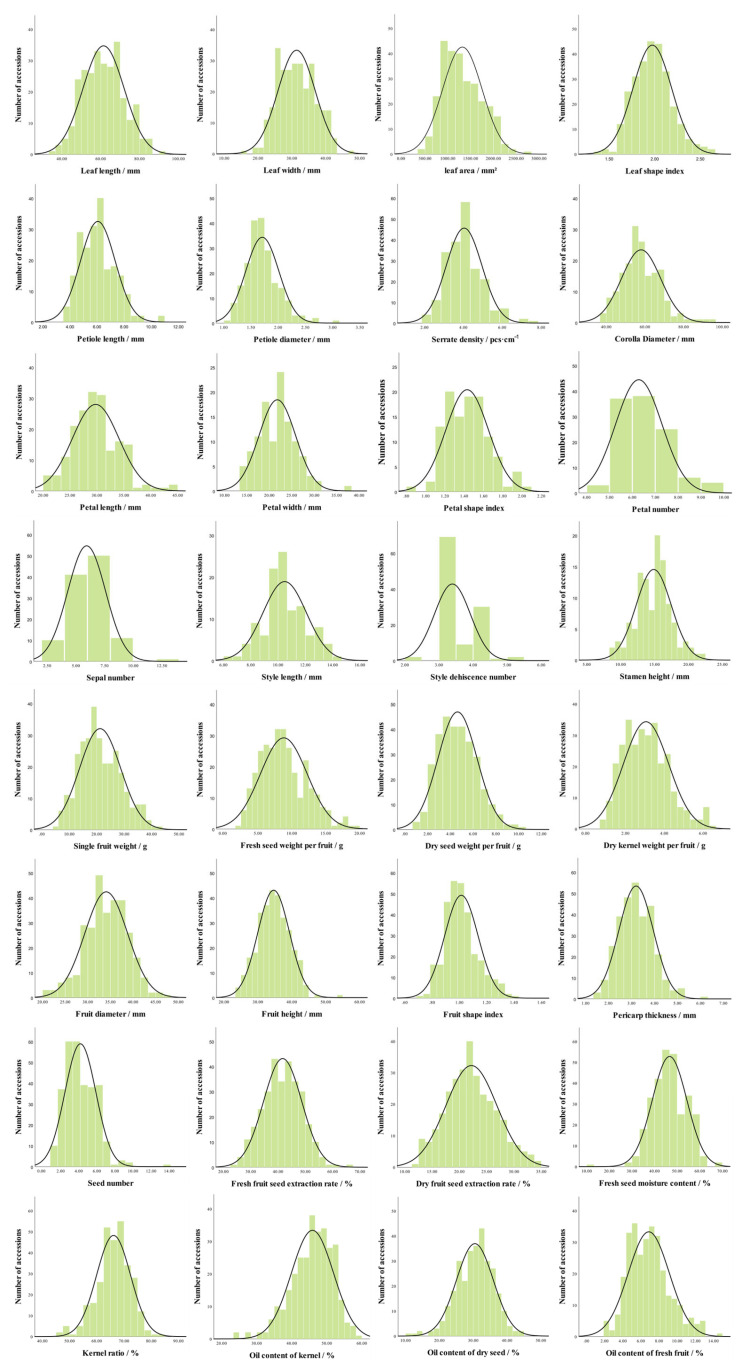
Frequency distribution of 32 quantitative traits of *C. oleiferas*.

In this study, the distributional characteristics of 32 quantitative traits were evaluated by the Kolmogorov-Smirnov normality test system (Figure 7, Table S1). The results showed that the two-tailed significance levels (Sig.) of 20 traits, such as leaf length and leaf width, were higher than 0.01, and the histograms of their frequency distributions showed a typical one-peak bell symmetrical pattern, which was consistent with the assumption of normal distribution. It is worth noting that although the original hypothesis was rejected in the K-S test for 9 traits, including leaf area and single fruit weight, their distributions still satisfied the single-peak nature and the absolute values of skewness and kurtosis were less than 1 (Table S1), so they could be categorized as approximate normal distribution. The remaining 3 traits, including petiole thickness, serration density and seed number showed significant non-normal characteristics, and their frequency distributions showed severe deviation patterns with extremely low two-tailed significance levels, so they did not conform to normal distribution.

#### 2.2.3. Pearson Correlation Analysis

**Figure 8:**
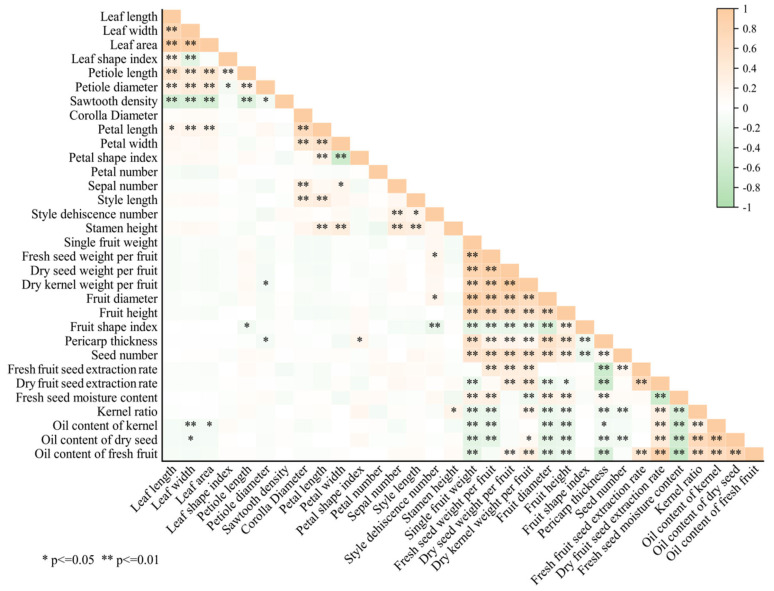
Correlation analysis of 32 quantitative traits in *C. oleifera*.

Pearson correlation analysis of 22 quantitative traits showed (Figure 8) that there were significant correlations among fruit traits, while the correlation of traits among leaf, flower, and fruit organs was weak, indicating that their genetic evolution was characterized by organ modularity, which was in line with the organ-independent evolution of plant phenotypic traits [24]. Among the leaf traits, leaf length and width, petiole length and thickness were all strongly and positively correlated with leaf area, suggesting that leaf size is a central characteristic of leaf phenotypic differentiation. Sawtooth density was negatively correlated with all leaf traits except the leaf shape index, implying that it is regulated by leaf size rather than shape. Among the flower traits, petal length and width, sepals number, and style length were all strongly positively correlated with flower diameter and weakly positively correlated with stamen height, suggesting that flower morphology is built primarily driven by overall size [25]. Petal number, on the other hand, was independent of the other traits, reflecting its neutral selective character in phenotypic evolution.

Single fruit weight was significantly correlated with other fruit traits except fresh seed yield, strongly positively correlated with fruit size indexes (e.g., fruit height, fruit diameter), and significantly negatively correlated with quality traits (e.g., oil content of seed kernels, dry seed rate), suggesting that fruit weight is a central hub for phenotypic differentiation and breeding evaluation [26,27]. Although the fruit shape index was strongly correlated with single fruit weight and fruit size, it was not significantly correlated with quality traits, suggesting that it is suitable for morphological classification rather than economic value assessment. The significant negative correlation of oil content of kernel and oil content of fresh fruit with single fruit weight and fruit size suggests the need to emphasize the balance between fruit size and oil accumulation in the selection of *C. oleifera* varieties [28].

#### 2.2.4. Principal Component Analysis (PCA)

**Figure 9:**
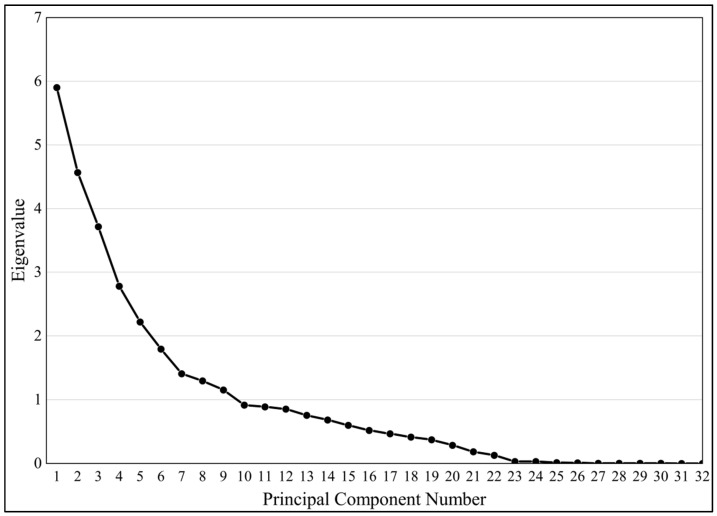
Principal component analysis scree plot.

PCA was performed on quantitative traits to simplify multidimensional data and focus on core traits. Based on “eigenvalues” > 1 [29] and combined with the scree plot (Table 2, Figure 9), the information embodied in the 32 traits was reduced to 7 principal components, which cumulatively explained 69.96% of the variance. The 1st principal component explained 18.44% of the variability and is called the fruit factor because the 1st principal component loadings were concentrated on fruit traits such as single fruit weight, fresh and dry seed weight per fruit, and fruit height. The variance contribution of the 2nd principal component was 14.27% and the dominant traits were economic indicators such as oil content of fresh fruit, oil content of dry seed, fresh fruit seed extraction rate, fresh seed moisture content, etc., so the 2nd principal component is called economic factor. The 3rd principal component explained 11.62% of the variability and the correlated traits were leaf traits such as leaf length and width, leaf area, petiole thickness, serration density, etc., known as leaf factor. The 4th principal component characterized floral traits such as petal length and width, flower diameter, number of petals, stamen height, etc. and is known as flower factor. The 5th principal component characterizes peel thickness, dry seed oil content, fresh seed yield, and petal number and is called the composite factor. Together, principal components 6 and 7 analyze the geometric anisotropic growth patterns of fruit shape, leaf shape, and petal shape indice, and are referred to as the shape factor.

The loading matrix analysis showed that 9 traits, including single fruit weight, fresh seed weight per fruit, oil content of fresh fruit and leaf area, had large loading values (Table 2), which can be used as core phenotypic markers with the functions of both genetic diversity characterization and phenotypic identification, and provide quantitative bases for the optimization of the evaluation system of *C. oleifera* germplasm resources.

#### 2.2.5. Determination of Ratings

In genetic analysis of quantitative traits, due to significant correlation between traits, variation in one quantitative trait may trigger correlated variation in other traits, which may lead to misclassification of specificity relationships [30]. Consequently, the aggregation of indicators demonstrating significant correlations in specificity testing was deemed methodologically justified. Based on integrative analysis combining Pearson correlation coefficients and principal component loadings, for the strong correlation traits that conformed to normal distribution (*p* ≤ 0.01), we established the following parameter groupings and selected representative traits: (1) leaf length and width, and petiole length and width were combined by leaf area; (2) petal length and width, and sepal number were combined by corolla diameter; (3) stamen height was combined by style length; (4) fruit height and diameter, fresh seed and dry seed and dry kernel weight per fruit were combined by single fruit weight; (5) dry fruit seed extraction rate and kernel ratio were combined by fresh fruit seed extraction rate; (6) oil content of dry seed was combined with that of kernel. Leaf, petal and fruit shape index were selected as fundamental descriptors of morphological configuration; oil content of fresh fruit was selected as the primary target trait for *C. oleifera* cultivation.

One-way analysis of variance (ANOVA) was carried out on leaf area, leaf shape index, flower diameter, petal shape index, style length, single fruit weight, fruit shape index, fresh fruit seed extraction rate, oil content of kernel and fresh fruit that conformed to normal distribution or approximately normal distribution. The LSD_0.05_ values were 51.167, 0.025, 1.493, 0.041, 0.296, 0.852, 0.014, 0.791, 0.681, and 0.256, respectively. In accordance with the UPOV classification method, the above-mentioned 10 traits were classified into 9, 9, 8, 5, 6, 9, 8, 9, 9, and 8 grades, respectively (Table 3). The sawtooth density did not conform to normal distribution, and the range classification method was employed, dividing it into 5 grades with a step size of 1.1. The number of petals and the style dehiscence number conformed to normal distribution, while the number of seeds did not conform to normal distribution, and since these three traits were only continuous on integers, they were classified into 6, 4, and 6 grades, respectively, in combination with the characteristics of data distribution (Table 3).

### 2.3. Comprehensive Evaluation of Economic Traits (Fruit Traits)

The TOPSIS comprehensive evaluation method provides an intuitive, concise and efficient way of evaluating multi-criteria decision-making to help decision-makers make optimal choices by comparing the distance between ideal and negative ideal solutions based on the ideal solution [31]. The TOPSIS comprehensive evaluation was conducted on 302 Ya’an *C. oleifera* germplasm resources for 16 fruit traits, including single fruit weight, fresh seed weight per fruit, pericarp thickness, and fresh fruit seed extraction rate (Table S2), from which 10 highest ranked resources were selected, including TQ122, TQ49, TQ219, TQ282, TQ109, TQ79, TQ135, TQ174, TQ55, TQ230 (Table 4). Nevertheless, it should be emphasized that the stability (i.e., reproducibility across years and geographic environments) of the screened superior germplasm has not been verified in this study due to its single-environment design. Trait stability is a key factor in determining the breeding value of germplasm resources and the regional adaptability of varieties [32], so future research needs to systematically assess the trait stability of these candidate materials through multi-point, multi-year regional trials combined with environmental genotyping (G × E).

## 3. Discussion

The germplasm resources of *C. oleifera* in Ya’an City exhibit distinctive variational characteristics in phenotypic traits, which reflect underlying genetic variability. The average diversity index and coefficient of variation of fruits are higher than those of flowers and leaves, which is in line with the rule that the economic traits of fruits are the evolutionary core of *C. oleifera* [33]. This also indicates that the traits related to fruits possess a higher potential for genetic improvement [34]. PCA further validates this point: the first principal component (18.44%) and the second principal component (14.27%) are dominated by fruit factors and economic factors respectively, which are highly consistent with the goal of “high yield and high oil content” in *C. oleifera* breeding [35]. It is worth noting that the strong negative correlation between the oil content of kernels and the weight of fruits suggests that the contradiction between yield and oil content needs to be balanced during the breeding process, which is in line with the findings of other studies on the “size-quality trade-off” of *C. oleifera* [33]. Additionally, the high conservation of petal color (99% white) in flower traits and the relatively high proportions of fully open flower morphology (81%) and wavy petal state (51%) may be related to its pollination mechanism [36,37], while the genetic diversity of fruit and seed traits reflects the diverse pressure of natural selection on reproductive strategies [38].

The 28 qualitative traits of the 302 *C. oleifera* germplasm resources in Ya’an were categorized into 2–5 grades according to their specific morphology, among which only leaf shape, fruit shape and seed shape were categorized into 5 grades, and the variation in organ shape was much larger than that of other traits [39], which is different from the studies in other regions, which is probably a result of the unique growth environment of *C. oleifera* in Ya’an and the combination of genetic factors [40]. The 8 qualitative traits of the flower were categorized into 2–3 grades, which manifested obvious distinctions from the grading of flower qualitative traits, particularly petal color, in the *Camellia* spp. testing guidelines. In accordance with the requirements of the DUS testing technical standards, the biological basis for determining variety specificity needs to be established on the basis of significant differences in phenotypic traits among populations [41]. However, in this study, the classification gradient of flower qualitative traits was inadequate, and the trait expressions presented a highly convergent distribution. Especially, the petal color was highly conserved genetically, making it difficult to support the determination of specificity. This further validated that the *Camellia* spp. testing guidelines could not be precisely applied to *C. oleifera*. While this study primarily focuses on the above-mentioned phenotypic traits, the observed variation provides indirect insights into potential genetic diversity. Future molecular analyses (e.g., SSR or SNP markers) are recommended to directly quantify genetic relationships among accessions.

The rating system of quantitative traits was constructed according to the principle of “trait independence” in UPOV guideline, 32 traits were integrated into 14 key traits including leaf area, leaf shape index, and weight of a single fruit by combining the strongly related traits, and the 13 traits were categorized into 5–9 grades by using the method of Least Significant Difference (LSD) and the Extreme Difference Method (EDM), which is more scientific and accurate than other methods for rating *C. oleifera* [42]. Among the 13 traits, 2 leaf traits were classified into 9 grades, and 5 flower traits were classified in the range of 4–8 grades, whereas the 6 fruit traits were classified into 8–9 grades except for the seed number which was classified into 6 grades, and the classification levels of the fruit and leaf traits were significantly higher than that of the flower traits, which was consistent with the lower grade differentiation characteristics shown in the flower qualitative traits, and further implied that the *C. oleifera* flowers have high genetic conservatism in phenotypic plasticity, and their morphological traits show strong genetic stability during phylogeny [43]. It is worth noting that the high coefficient of variation in seed number and the low level of differentiation may be a result of the special genetic regulation mechanism of *C. oleifera* and environmental disturbances, and a manifestation of the “quantity-quality” trade-off strategy [44].

As an important woody oil seed crop, economic traits such as fruit weight and seed yield are the core evaluation parameters of *C. oleifera* yield and quality, which directly affect its economic value and breeding potential [45]. The 10 highest scoring resources including TQ122, TQ49, TQ219, TQ282, TQ109, TQ79, TQ135, TQ174, TQ55, TQ230 were screened out among 302 resources by TOPSIS comprehensive evaluation method, and the results showed that high scoring germplasm needs to combine both trait specificity and comprehensive trait advantages. TQ122, for example, which ranked first in the overall ranking, had a single fruit weight (24.78 g) and peel thickness (2.78 mm) that were not significantly different from the mean values (21.22g, 3.20mm), but its fresh fruit seed extraction rate (52.09%), oil content of kernel (53.66%) and oil content of fresh fruit (12.27%) were significantly better than the group mean (6.87%), showing a synergistic advantage of multi-traits. On the contrary, although TQ291 had the largest single fruit weight (43.88 g), the key indexes such as pericarp thickness (4.33 mm), dry fruit seed extraction rate (16.81%), oil content of kernel (27.33%) and oil content of fresh fruit (2.07%) were close to the worst values, which caused its comprehensive ranking to drop to 158th place, and also proved the negative correlation between fruit weight and oil content. This phenomenon may result from a resource allocation trade-off strategy during *C. oleifera* fruit development: fruit expansion mainly relies on carbohydrate accumulation, while oil synthesis consumes a large amount of carbon and energy, the allocation of large-fruited germplasm resources is biased toward the construction of pericarp and seed shell morphology, crowding out the supply of precursors for oil synthesis (e.g., acetyl-CoA, glycerol), and decreasing the efficiency of seed oil conversion [46]. At the same time, its thick pericarp increases the proportion of non-oil tissues, dilutes the oil content of the whole fruit, and may impede the translocation of photosynthetic products to the seed kernel [47]. Genes regulating fruit size and oil synthesis genes may be antagonistically expressed, and similar mechanisms have been demonstrated in oil palm [48]. This negative correlation is the main obstacle to the simultaneous improvement of high yield and high oil content in *C. oleifera*. Selection based solely on fruit weight leads to a significant deterioration in key oil characteristics (oil content, seed yield, and fruit skin thickness), thereby reducing overall economic value. Therefore, the results of this study strongly support the use of a multi-trait synergistic selection strategy for screening superior germplasm, abandoning the traditional approach of solely pursuing the heaviest fruit weight, and instead focusing on achieving a good balance across multiple core economic traits to avoid the trait trade-off effects associated with single-trait selection [49].

## 4. Materials and Methods

### 4.1. Experimental Materials

A total of 302 *C. oleifera* germplasm resources randomly collected throughout Ya’an City were used in this study, and all the materials were centrally planted in the *C. oleifera* germplasm resource base in Tianquan County, Ya’an City, and all the samples were more than 8 years old and managed uniformly.

### 4.2. Measurement of Phenotypic Traits

The above 302 *C. oleifera* germplasm resources were measured for four consecutive years (from 2021 to 2024) and averaged to weaken annual environmental fluctuations. During the fruit ripening period of *C. oleifera*, 30 ripe fruits of *C. oleifera* trees were randomly collected (all of them were collected if less than 30), 20 open flowers and ripe leaves, the phenotypic traits of flowers and branches were observed and determined in the field, and the leaves and fruits were brought back to the laboratory in a 4 °C freshness-keeping box to be determined for the phenotypic traits.

In this study, based on the Technical regulations for investigating and catalogue of genetic resources on *Camellia* spp. and modifying it with the characteristics of common *C. oleifera* [7], 28 typical qualitative traits covering the key organs such as branches, leaves, flowers and fruits were chosen, these traits are the primary basis for determining Distinctness in the DUS test, including 4 branch traits: branch posture, juvenile shoot coloration, number of terminal bud and bud pubescence; 6 leaf traits: leaf shape, leaf vein state, leaf surface morphology, leaf tip morphology, leaf base morphology and leaf serration morphology; 8 flower traits: flower opening morphology, flower color, petal shape, degree of petal apex dehiscence, petal surface morphology, degree of style dehiscence, filaments connate and ovary hairs length; 8 fruit traits, fruit shape, fruit angularity, fruit navel, fruit pedicel, fruit color, Fruit surface condition, seed color and seed shape; tree life form and canopy form. The 32 quantitative traits mainly focused on fruit yield traits such as single fruit weight, fresh and dry seed weight per fruit, seed number, as well as fruit economic traits such as fresh and dry seed rate, oil content of kernel, oil content of fresh fruit, which are the key indexes for evaluating the value of *C. oleifera* germplasm resources and breeding selection.

Corolla diameter, petal length, petal width, style length, stamen height, leaf length, leaf width, petiole length, petiole diameter, fruit height, fruit diameter, pericarp thickness of 302 *C. oleifera* germplasm resources were measured using vernier calipers with a precision of 0.01 mm, and the number of petals, sepals, and the number of stylar dehiscence were observed by visual inspection. Fruit weight, fresh seed weight, dry seed weight, and dry kernel weight were measured accurately for each fruit using a 0.01 g electronic balance. Oil content of kernel using a nuclear magnetic resonance oil content meter (Zhejiang topu yunnong Technology Co., Ltd., Hangzhou, China). Based on the results, other traits were calculated: leaf shape index = leaf length/leaf width, leaf area = 2/3 leaf length × leaf width, fruit shape index = fruit height/fruit diameter, petal index = petal length/petal width; Fresh seed rate = fresh seed weight/fruit weight × 100%, dry seed rate = dry seed weight/fruit weight × 100%, Kernel ratio = dry kernel weight/fruit weight × 100%, fresh seed moisture content = (fresh seed weight − dry seed weight)/fresh seed weight × 100%, oil content of dry seed = kernel ratio × oil content of kernel, oil content of fresh fruit = dry seed rate × kernel-fruit ratio × oil rate of kernel.

### 4.3. Statistical Analysis

Trait values are the mean of four measurements from 2021 to 2024. Phenotypic diversity of 28 qualitative traits was assessed by calculating frequency distributions, and the Shannon-Wiener index (H′) was calculated by $H^{\prime }=-\sum_{i=0}^{n}P_{i}\ln P_{i}$. Maximum, minimum, standard deviation (SD), median, mean and coefficient of variation (CV, %) were calculated for 32 quantitative traits. Data were processed using Excel 2021, Pearson’s correlation analysis, principal component analysis (PCA) and normality test were performed using Origin 2021b software, and TOPSIS analysis was performed using SPSS software v22.0. The least Significant Difference (LSD) is used to grade quantitative traits that conform to a normal distribution. The method takes the average value as the center and the two sides are equidistant 2 times LSD0.05 [50]. The range method is used to grade traits that are not normally distributed, with the formula y = G ± (1/2 + n)x (n = 0, 1, 2, 3, 4, G is the median of the data, and x is the graded variance) [18].

## 5. Conclusions

In this study, we systematically analyzed the phenotypic diversity of 302 *C. oleifera* germplasm resources in Ya’an, Sichuan Province. For the first time, it was clarified that Ya’an *C. oleifera* germplasm showed a phenotypic diversity gradient of “fruit > leaf > flower” at the organ level, and its high variability in economic traits provided a direct basis for directional breeding for high yield and high oil. Through correlation analysis and principal component analysis, 28 qualitative traits were categorized into 2–5 grades; meanwhile, 32 quantitative traits were combined into 13 independent indexes and further subdivided into 4–9 grades, and a multidimensional grading system covering both qualitative and quantitative traits was constructed, which provided quantifiable and reproducible phenotypic evaluation standards for the formulation of DUS test guidelines for *C. oleifera*, and significantly compensates for the deficiencies of existing broad DUS guidelines in the characterization and quantitative grading of *C. oleifera*-specific traits. Based on the TOPSIS multidimensional evaluation model, 10 germplasm resources with excellent comprehensive performance, including TQ122, TQ49 and TQ219, were screened. It is worth noting that these superior plants generally showed synergistic advantages of key economic traits rather than extreme performance of a single trait, which provided core materials and multi-trait synergistic optimization for the collaborative breeding of “high yield and high oil” in *C. oleifera*.

In the future, we will integrate molecular marker technologies such as SSR and SNP to genotype core germplasm, analyze the genetic basis of excellent phenotypes and establish phenotype-genotype associations, and accelerate molecular marker-assisted breeding; we will carry out multi-year multi-point testing of superior strains in the main distribution areas of *C. oleifera* and systematically evaluate the genotype-environment interactions (G × E) and the stability of economic traits such as yield and oil content, and screen At the same time, by combining transcriptome, metabolome and other multi-omics technologies, we will analyze the key regulatory pathways and genes of oil synthesis in high oil germplasm, so as to provide theoretical targets for genetic improvement.

## Figures and Tables

**Table 1:** Frequency distribution and grading evaluation of 28 qualitative traits in *Camellia oleifera*.

	Trait	Frequency Distribution/%					H′
		1	2	3	4	5	
Plant	Life form	45.70	54.30				0.69
	Tree form	34.11	56.29	9.60			0.92
Branch	Branch posture	12.25	54.64	28.48	4.64		1.09
	Juvenile shoot coloration	84.11	15.89				0.44
	Number of terminal bud	55.30	8.94	35.76			0.91
	Bud pubescence	7.62	92.38				0.27
Leaf	Leaf shape	10.60	52.32	20.53	7.62	8.94	1.31
	Leaf vein state	90.73	9.27				0.31
	Leaf surface morphology	58.28	37.75	3.97			0.81
	Leaf tip morphology	48.34	21.52	4.64	25.50		1.17
	Leaf base morphology	19.87	50.99	18.87	10.26		1.21
	Leaf serration morphology	5.63	55.30	12.58	26.49		1.10
Fower	Flower opening pattern	13.91	81.13	4.97			0.59
	Flower color	99.01	0.99				0.06
	Petal shape	26.49	50.00	23.51			1.04
	Degree of petal apex dehiscence	6.95	89.07	3.97			0.42
	Petal surface morphology	34.44	50.99	14.57			0.99
	Degree of style dehiscence	56.29	25.83	17.88			0.98
	Filaments connection	62.25	37.75				0.66
	Ovary hair length	29.80	70.20				0.61
Fruit	Fruit shape	13.91	47.68	12.25	21.52	4.64	1.36
	Fruit angularity	55.30	44.70				0.69
	Fruit surface condition	73.18	26.82				0.58
	Fruit pedicel	16.89	38.74	44.37			1.03
	Fruit navel	6.29	50.99	42.72			0.88
	Pericarp color	33.77	59.27	6.62	0.33		0.88
	Seed color	31.46	21.52	37.09	9.93		1.29
	Seed shape	20.20	4.97	20.53	40.07	14.24	1.44
average							0.85

Note: Grade descriptions of the 3 qualitative traits not shown in the figure belows. Leaf vein state: (1) Concavity, (2) Even; Leaf surface morphology: (1) Even, (2) Wavy, (3) Revolute; Petal surface morphology: (1) Even, (2) Wavy, (3) Revolute.

**Table 2:** Principal component analysis loading matrix for 32 quantitative traits in *C. oleifera*.

Traits	Eigenvector of the Principal Component						
	1	2	3	4	5	6	7
Leaf length	−0.434	−0.265	0.695	−0.236	−0.088	0.114	0.208
Leaf width	−0.307	−0.159	0.801	−0.169	−0.031	−0.078	−0.329
Leaf area	−0.402	−0.234	0.790	−0.217	−0.055	0.026	−0.051
Leaf shape index	−0.177	−0.139	−0.162	−0.073	−0.082	0.275	0.740
Petiole length	−0.219	−0.209	0.483	−0.308	−0.039	0.367	0.160
Petiole diameter	−0.186	−0.319	0.454	−0.162	0.013	0.356	−0.003
Sawtooth density	0.248	0.250	−0.432	0.093	0.196	0.084	−0.007
Corolla diameter	−0.147	−0.090	0.323	0.693	0.100	−0.022	0.177
Petal length	−0.241	−0.010	0.459	0.549	0.263	−0.139	0.116
Petal width	−0.107	0.168	0.153	0.710	0.100	0.343	0.042
Petal shape index	−0.083	−0.223	0.239	−0.355	0.230	−0.539	0.147
Petal number	0.028	−0.071	−0.174	0.008	−0.432	0.105	0.246
Sepal number	0.118	0.127	0.191	0.494	0.051	−0.041	−0.008
Style length	0.015	0.055	0.228	0.461	0.161	0.003	0.062
Style dehiscence number	0.340	0.027	0.036	0.310	0.022	0.167	0.222
Stamen height	−0.103	0.184	0.273	0.488	0.321	−0.094	−0.064
Single fruit weight	0.907	−0.190	0.127	−0.089	0.183	0.085	−0.001
Fresh seed weight per fruit	0.929	0.045	0.257	−0.033	−0.126	0.019	0.026
Dry seed weight per fruit	0.890	0.278	0.282	−0.087	−0.046	0.006	0.020
Dry kernel weight per fruit	0.813	0.392	0.342	−0.119	0.057	−0.051	0.014
Fruit diameter	0.856	−0.275	0.142	−0.055	0.223	0.106	−0.099
Fruit height	0.626	−0.172	0.144	−0.059	0.128	−0.474	0.342
Fruit shape index	−0.328	0.123	−0.019	−0.006	−0.130	−0.620	0.483
Pericarp thickness	0.382	−0.500	−0.005	−0.136	0.614	−0.013	0.101
Seed number	0.521	0.027	0.352	−0.038	−0.151	0.275	0.225
Fresh seed rate	0.255	0.519	0.296	0.117	−0.664	−0.110	0.004
Dry seed rate	0.096	0.849	0.264	−0.012	−0.351	−0.081	−0.015
Fresh seed moisture content	0.150	−0.751	−0.035	0.164	−0.248	−0.014	0.040
Kernel ratio	−0.238	0.483	0.241	−0.156	0.428	−0.213	−0.036
Oil content of kernel	−0.162	0.518	−0.157	−0.282	0.326	0.397	0.222
Oil content of dry seed	−0.256	0.674	0.024	−0.294	0.499	0.157	0.133
Oil content of fresh fruit	−0.080	0.943	0.199	−0.158	0.020	0.028	0.053
Eigenvalue	5.900	4.565	3.717	2.781	2.220	1.795	1.409
Contribution rate/%	18.438	14.267	11.617	8.692	6.937	5.611	4.403
Cumulative contribution rate/%	18.438	32.705	44.322	53.014	59.951	65.561	69.964

**Table 3:** Grade evaluation of 14 flower, leaf, and fruit traits in *C. oleifera*.

Trait	Rating Range									LSD_0.05_	Step
	1	2	3	4	5	6	7	8	9		
Leaf area/mm^2^	<790	790–1000	1000–1210	1210–1420	1420–1630	1630–1840	1840–2050	2050–2260	≥2260	51.167	
Leaf shape index	<1.6	1.6–1.7	1.7–1.8	1.8–1.9	1.9–2.0	2.0–2.1	2.1–2.2	2.2–2.3	≥2.3	0.025	
Corolla diameter/mm	<43	43–46	49–55	55–61	61–67	67–73	73–79	≥79		1.493	
Petal shape index	<1.1	1.1–1.3	1.3–1.5	1.5–1.7	≥1.7					0.041	
Style length/mm	<7.4	7.4–8.6	8.6–9.8	9.8–11.0	11.0–12.2	≥12.2				0.296	
Single fruit weight/g	<12.2	12.2–15.8	15.8–19.4	19.4–23.0	23.0–26.6	26.6–30.2	30.2–33.8	33.8–37.4	≥37.4	0.852	
Fruit shape index	<0.79	0.85–0.91	0.91–0.97	0.97–1.03	1.03–1.09	1.09–1.15	1.15–1.21	≥1.21		0.014	
Fresh fruit seed extraction rate/%	<30.4	30.4–33.6	33.6–36.8	36.8–40.0	40.0–43.2	43.2–46.4	46.4–49.6	49.6–52.8	≥52.8	0.791	
Oil content of kernel/%	<36.2	36.2–39.0	39.0–41.8	41.8–44.6	44.6–47.4	47.4–50.2	50.2–53.0	53.0–55.8	≥55.8	0.681	
Oil content of fresh fruit/%	<3.7	3.7–5.0	5.0–6.3	6.3–7.6	7.6–8.9	8.9–10.2	10.2–11.5	≥11.5		0.256	
serrate density/pcs·cm^−1^	<2.35	2.35–3.45	3.45–4.55	4.55–5.65	≥5.65						1.1
Number of petals	4	5	6	7	8	≥9					1
Style cracking number	1	2	3	4							1
Seed number	<3	3–5	5–7	7–9	9–11	≥11					2

**Table 4:** Scores and ranking of 10 germplasm resources screened from 302 *C. oleifera* germplasm resources by TOPSIS.

Number	Distance to Positive Ideal Solution(D+)	Distance to Negative Ideal Solution(D−)	Relative Closeness(C)	Rank
TQ122	0.166	0.470	0.739	1
TQ49	0.170	0.472	0.735	2
TQ219	0.175	0.464	0.726	3
TQ282	0.178	0.471	0.725	4
TQ109	0.182	0.477	0.724	5
TQ79	0.189	0.470	0.714	6
TQ135	0.189	0.466	0.712	7
TQ174	0.190	0.465	0.710	8
TQ55	0.190	0.455	0.706	9
TQ230	0.190	0.447	0.702	10

## Data Availability

The data provided in this study are available upon request from the corresponding author. Due to privacy concerns, these data are not publicly available.

## Supplementary Materials

The following supporting information can be downloaded at: https://www.mdpi.com/article/10.3390/plants14142249/s1, Table S1. K-S text for normality of 32 quantitative traits in *C. oleifera*. Table S2. Scores and ranking of 302 *C. oleiferas* germplasm resources by TOPSIS.

## References

[1] Zhao D.W., Hodkinson T.R., Parnell J.A.N. (2023). Phylogenetics of global Camellia (Theaceae) based on three nuclear regions and its implications for systematics and evolutionary history. J. Syst. Evol..

[2] Quan W., Wang A., Gao C., Li C. (2022). Applications of Chinese *Camellia oleifera* and its By-Products: A Review. Front. Chem..

[3] He J., Wu X., Yu Z. (2021). Microwave pretreatment of camellia (*Camellia oleifera* Abel.) seeds: Effect on oil flavor. Food Chem..

[4] Luan F., Zeng J., Yang Y., He X., Wang B., Gao Y., Zeng N. (2020). Recent advances in *Camellia oleifera* Abel: A review of nutritional constituents, biofunctional properties, and potential industrial applications. J. Funct. Foods.

[5] Hu Y.L. (2019). Discussion on influencing factors and reform scheme of *Camellia oleifera* industry supply side. South China For. Sci..

[6] Cheng J., Jiang D., Cheng H., Zhou X., Fang Y., Zhang X., Xiao X., Deng X., Li L. (2018). Determination of *Camellia oleifera* Abel. germplasm resources of genetic diversity in China using ISSR Markers. Not. Bot. Horti Agrobot. Cluj-Napoca.

[7] (2023). Technical Regulations for Investigating and Catalogue of Genetic Resources on *Camellia* spp..

[8] Chen L., Zhou J. (2015). Different physical properties of summer precipitation clouds over Qinghai-Xizang plateau and Sichuan Basin. Plateau Meteorol..

[9] He Z., Liu C., Wang X., Wang R., Chen Y., Tian Y. (2020). Assessment of genetic diversity in *Camellia oleifera* Abel. accessions using morphological traits and simple sequence repeat (SSR) markers. Breed. Sci..

[10] Zhu Y., Liang D., Song Z., Tan Y., Guo X., Wang D. (2022). Genetic diversity analysis and core germplasm collection construction of *Camellia oleifera* based on fruit phenotype and SSR data. Genes.

[11] Liu N., Lyu X., Zhang X., Zhang G., Zhang Z., Guan X., Chen X., Yang X., Feng Z., Gao Q. (2024). Reference genome sequence and population genomic analysis of peas provide insights into the genetic basis of Mendelian and other agronomic traits. Nat. Genet..

[12] Zhang M., Liu Y.H., Xu W., Smith C.W., Murray S.C., Zhang H.B. (2020). Analysis of the genes controlling three quantitative traits in three diverse plant species reveals the molecular basis of quantitative traits. Sci. Rep..

[13] Verma H., Borah J.L., Sarma R.N. (2019). Variability Assessment for Root and Drought Tolerance Traits and Genetic Diversity Analysis of Rice Germplasm using SSR Markers. Sci. Rep..

[14] UPOV (1991). Union Internationale Pour la Protection des Obtentions Végétales.

[15] (2016). Guidelines for the Conduct of Tests for Distinctness, Uniformity and Stability (DUS)-Oil-Tea Camellia (*Camellia* spp.).

[16] Liu Y.F., Zhang J.H., LÜ B., Yang X.H., Li Y.G., Wang Y., Wang J.M., Zhang H., Guan J.J. (2013). Statistic analysis on quantitative characteristics for developing the DUS test guideline of *Ranunculus asiaticus* L.. J. Integr. Agric..

[17] Wu Q.C., Zhang D., Zhang Q., Zang D.K. (2019). Development of DUS Test Guidelines for new varieties of *Viburnum* L.. J. For. Res..

[18] Wang Y., Hu G.P., Liu Z.S., Zhang J., Ma L., Tian T., Wang H., Chen T., Chen Q., He W. (2022). Phenotyping in flower and main fruit traits of Chinese cherry [*Cerasus pseudocerasus* (Lindl.) G. Don]. Sci. Hortic..

[19] Chen T.L., Chen G.Q., Lu L., Lan H.Q., Fan Y.H., Ling P. (2025). Genetic Diversity Analysis of Phenotypic Traits in Seedling *Camellia oleifera* on Hainan Island. Chin. J. Trop. Crops.

[20] Zhou Y.L. (2022). Evaluation of Main Characters of Ya’an Wild *Camellia oleifera* and Screening of Excellent Trees. Master’s Thesis.

[21] Zhou X., Wang Z.D., Li J., Xu H., Peng X.Y., Zhu J.M. (2024). Diversity analysis of phenotypic traits of wild *Camellia oleifera* in Shangcheng County. J. Xinyang Agric. For. Univ..

[22] Xu J.J., Zhu Y.Y., Wang G. (2021). Comprehensive evaluation and Pphenotypic diversity analysis of *Camellia meiocarpa* in Guizhou. J. Zhejiang For. Sci. Technol..

[23] Wu Y., Zhang L., Zhang Y., Zhou H., Ma L. (2024). Roles of antioxidant enzymes, secondary metabolites, and lipids in light adaption of tea-oil plant (*Camellia oleifera* Abel). J. Plant Growth Regul..

[24] Elizabeth F. (2016). Plant Systematics: A Phylogenetic Approach. Rhodora.

[25] Jules S., Marie-Laure N., Eric G. (2019). Reproductive phenology as a dimension of the phenotypic space in 139 plant species from the mediterranean. New Phytol..

[26] Gecer M.K., Kan T., Gundogdu M., Ercisli S., Ilhan G., Sagbas H.I. (2020). Physicochemical characteristics of wild and cultivated apricots (*Prunus armeniaca* L.) from Aras valley in Turkey. Genet. Resour. Crop Evol..

[27] Yang L., Gao C., Xie J.J., Qiu J., Deng Q., Zhou Y.C., Liao D., Deng C. (2022). Fruit economic characteristics and yields of 40 superior *Camellia oleifera* Abel plants in the low-hot valley area of Guizhou Province, China. Sci. Rep..

[28] López-Bernal Á., Fernandes-Silva A.A., Vega V.A., Hidalgo J.C., León L., Testi L., Villalobos F.J. (2021). A fruit growth approach to estimate oil content in olives. Eur. J. Agron..

[29] Chan Y.H. (2024). Biostatistics 302. Principal component and factor analysis. Singap. Med. J..

[30] Clo J., Opedal Ø.H. (2021). Genetics of quantitative traits with dominance under stabilizing and directional selection in partially selfing species. Evolution.

[31] Azadi A., Jalali A.S., Navidi M.N. (2023). Land evaluation approaches comparing TOPSIS and SAW with parametric methods for rice cultivation. Environ. Monit. Assess..

[32] Samuel Cristian D., Andrei Daniel Z., Leomar Guilherme W., Anderson Simionato M., Rodrigo Z., Josiane C., Giovani B. (2019). Across Year and Year-by-year GGE Biplot Analysis to Evaluate Soybean Performance and Stability in Multi-Environment Trials. Euphytica.

[33] Zhang F.H., Li Z., Zhou J.Q., Gu Y.Y., Tan X.F. (2021). Comparative study on fruit development and oil synthesis in two cultivars of *Camellia oleifera*. BMC Plant Biol..

[34] Wang J.T., Ye H., Zhou H.J., Chen P.P., Liu H.Z., Xi R.M., Wang G., Hou N., Zhao P. (2022). Genome-wide association analysis of 101 accessions dissects the genetic basis of shell thickness for genetic improvement in Persian walnut (*Juglans regia* L.). BMC Plant Biol..

[35] Wang H., Liu Y.H., Song J.M., Deng Y.J., Zhou Y., Lai H.G. (2022). Agronomic traits and quality screening of superior *Camellia oleifera* in tropical areas. Tree Genet. Mol. Breed..

[36] Trunschke J., Lunau K., Pyke G.H., Ren Z.X., Wang H. (2021). Flower color evolution and the evidence of pollinator-mediated selection. Front. Plant Sci..

[37] Cerović R., Fotirić Akšić M., Meland M. (2020). Success rate of individual pollinizers for the pear cultivars “Ingeborg” and “Celina” in a Nordic climate. Agronomy.

[38] Cutter A.D. (2019). Reproductive transitions in plants and animals: Selfing syndrome, sexual selection and speciation. New Phytol..

[39] Xing K.F., Zhang J., Chen S., Zhang L.D., Xie H.X., Zhao Y., Rong J. (2024). Diversity analysis of phenotypic traits and comprehensive evaluation of *Camellia oleifera* excellent germplasm resources. J. Zhejiang Univ. (Agric. Life Sci.).

[40] Wu S., Zhang B., Keyhaninejad N., Rodríguez G.R., Kim H.J., Chakrabarti M., Illa-Berenguer E., Taitano N.K., Gonzalo M.J., Díaz A. (2018). A common genetic mechanism underlies morphological diversity in fruits and other plant organs. Nat. Commun..

[41] UPOV (2022). General Introduction to the Examination of Distinctness, Uniformity and Stability and the Development of Harmonized Descriptions of New Varieties of Plants: TG/1/3.

[42] Chen T., Liu L., Zhou Y.L., Zheng Q., Luo S.Y., Xiang T.T., Zhou L.J., Feng S.L., Yang H.Y., Ding C.B. (2023). Characterization and comprehensive evaluation of phenotypic characters in wild *Camellia oleifera* germplasm for conservation and breeding. Front. Plant Sci..

[43] Reid J.M., Acker P. (2022). Properties of phenotypic plasticity in discrete threshold traits. Evolution.

[44] Opedal Ø.H., Armbruster W.S., Hansen T.F., Holstad A., Pélabon C., Andersson S., Campbell D.R., Caruso C.M., Delph L.F., Eckert C.G. (2023). Evolvability and trait function predict phenotypic divergence of plant populations. Proc. Natl. Acad. Sci. USA.

[45] Tu J., Chen J.F., Zhou J.H., Ai W.S., Chen L.S. (2019). Plantation Quality Assessment of *Camellia oleifera* in Mid-Subtropical China. Soil Tillage Res..

[46] Falchi R., Bonghi C., Drincovich M.F., Famiani F., Lara M.V., Walker R.P., Vizzotto G. (2020). Sugar Metabolism in Stone Fruit: Source-Sink Relationships and Environmental and Agronomical Effects. Front. Plant Sci..

[47] Shen C.H., Chen R.F., Liu Z. (2024). Characteristics of the transport and distribution of photosynthetic products during fruit expansion of *Camellia gauchowensis*. J. Cent. South Univ. For. Technol..

[48] Martin J.J.J., Xu W., Li X., Liu X., Zhou L., Li R., Fu D., Li Q., Cao H. (2025). Deciphering the Molecular Mechanisms of Oil Palm Lipid Metabolism Through Combined Metabolomics and Transcriptomics. Food Chem..

[49] Zhang Z.J., Meng J.X., Pan D.F., Yang C., Li Y. (2018). Mating system and progeny genetic diversity of *Camellia oleifera* ‘Ruan Zhi’. J. For. Res..

[50] UPOV (2016). Guidelines for the Conduct of Tests for Distinctness, Uniformity and Stability of Sweet Cherry: TG/35/7.

