# Supplementary material for: Phenotypic Diversity Analysis and Integrative Evaluation of Camellia oleifera Germplasm Resources in Ya’an, Sichuan Province
Source: Plants (Basel). 2025 Jul 21;14(14):2249. doi: 10.3390/plants14142249 (PMC12298781; doi:10.3390/plants14142249)
Supplement: Supplementary file 1 [file plants-14-02249-s001.zip › Table S1.pdf]

Table S1. K-S text for normality of 32 quantitative traits in *C. oleifera*

Traits	Range			K-S value	Sig.value	Skewness	Kurtosis
	Absolute value	Positive	Negative				
Leaf length	0.044	0.044	-0.02 5	0.044	0.200	-0.417	0.115
Leaf width	0.051	0.051	-0.034	0.051	0.083	-0.543	0.144
Leaf area	0.076	0.076	-0.040	0.076	0.001	0.447	-0.406
Leaf shape index	0.044	0.044	-0.025	0.044	0.200	0.238	0.289
Petiole length	0.062	0.062	-0.040	0.062	0.049	0.900	0.696
Petiole diameter	0.096	0.096	-0.044	0.096	0.000	2.246	1.025
Sawtooth density	0.103	0.103	-0.048	0.103	0.000	1.047	0.733
Corolla Diameter	0.068	0.068	-0.030	0.068	0.042	1.170	0.710
Petal length	0.060	0.060	-0.028	0.060	0.200	0.586	0.534
Petal width	0.054	0.054	-0.036	0.054	0.200	0.627	0.432
Petal shape index	0.056	0.056	-0.047	0.056	0.200	0.223	0.437
Petal number	0.184	0.184	-0.077	0.184	0.000	0.156	0.733
Sepal number	0.096	0.096	-0.076	0.096	0.013	0.561	0.413
Style length	0.067	0.067	-0.050	0.067	0.200	-0.139	0.134
Style dehiscence number	0.286	0.286	-0.210	0.286	0.000	0.265	0.653
Stamen height	0.063	0.056	-0.063	0.063	0.200	0.070	0.063
Single fruit weight	0.065	0.065	-0.033	0.065	0.003	-0.288	0.380
Fresh seed weight per fruit	0.060	0.060	-0.037	0.060	0.010	-0.034	0.543
Dry seed weight per fruit	0.053	0.053	-0.034	0.053	0.043	-0.111	0.436
Dry kernel weight per fruit	0.060	0.060	-0.037	0.060	0.011	-0.033	0.536
Fruit diameter	0.035	0.023	-0.035	0.035	0.200	0.094	-0.204
Fruit height	0.024	0.024	-0.020	0.024	0.200	0.391	0.236
Fruit shape index	0.084	0.084	-0.040	0.084	0.000	0.406	0.608
Pericarp thickness	0.049	0.049	-0.027	0.049	0.077	0.443	0.378
Seed number	0.068	0.068	-0.038	0.068	0.002	3.321	0.989
Fresh fruit seed extraction rate	0.028	0.027	-0.028	0.028	0.200	0.022	0.115
Dry fruit seed extraction rate	0.043	0.043	-0.021	0.043	0.200	-0.229	0.245
Fresh seed moisture content	0.036	0.033	-0.036	0.036	0.200	0.606	-0.137
Kernel ratio	0.066	0.028	-0.066	0.066	0.003	0.655	-0.530
Oil content of kernel	0.060	0.043	-0.060	0.060	0.010	0.709	-0.692
Oil content of dry seed	0.042	0.023	-0.042	0.042	0.200	0.480	-0.361
Oil content of fresh fruit	0.055	0.055	-0.043	0.055	0.029	0.282	0.583
