# Supplementary material for: Phenotypic Diversity Analysis and Integrative Evaluation of Camellia oleifera Germplasm Resources in Ya’an, Sichuan Province
Source: Plants (Basel). 2025 Jul 21;14(14):2249. doi: 10.3390/plants14142249 (PMC12298781; doi:10.3390/plants14142249)
Supplement: Supplementary file 1 [file plants-14-02249-s001.zip › Table S2.pdf]

Table S2 Scores and ranking of 302 *C. oleiferas* germplasm resources by TOPSIS

Number	Distance to positive ideal solution(D+)	Distance to negative ideal solution(D-)	Relative closeness(C)	Rank
TQ1	0.258	0.43	0.625	65
TQ2	0.325	0.361	0.527	254
TQ3	0.381	0.314	0.452	294
TQ4	0.215	0.448	0.676	15
TQ5	0.247	0.441	0.641	50
TQ6	0.287	0.351	0.551	222
TQ7	0.262	0.372	0.586	150
TQ8	0.241	0.48	0.666	20
TQ9	0.339	0.348	0.507	269
TQ10	0.255	0.362	0.586	151
TQ11	0.236	0.427	0.644	48
TQ12	0.348	0.343	0.496	278
TQ13	0.355	0.35	0.497	276
TQ14	0.333	0.379	0.532	251
TQ15	0.266	0.414	0.608	99
TQ16	0.266	0.395	0.598	122
TQ17	0.241	0.438	0.645	46
TQ18	0.223	0.428	0.657	31
TQ19	0.222	0.405	0.646	45
TQ20	0.241	0.452	0.652	41
TQ21	0.242	0.409	0.628	62
TQ22	0.26	0.395	0.603	107
TQ23	0.219	0.423	0.659	25
TQ24	0.197	0.452	0.696	11
TQ25	0.282	0.428	0.603	108
TQ26	0.28	0.417	0.598	123
TQ27	0.221	0.451	0.671	18
TQ28	0.23	0.429	0.651	43
TQ29	0.285	0.403	0.586	152
TQ30	0.248	0.397	0.616	80
TQ31	0.284	0.388	0.577	175
TQ32	0.359	0.333	0.482	282
TQ33	0.347	0.328	0.486	280
TQ34	0.394	0.34	0.464	289
TQ35	0.248	0.397	0.615	81
TQ36	0.233	0.388	0.624	67
TQ37	0.344	0.342	0.498	275
TQ38	0.296	0.418	0.586	153
TQ39	0.284	0.397	0.583	161
TQ40	0.267	0.418	0.61	92
TQ41	0.266	0.424	0.614	84

TQ42	0.315	0.366	0.538	243
TQ43	0.306	0.383	0.556	209
TQ44	0.351	0.341	0.493	279
TQ45	0.3	0.336	0.529	253
TQ46	0.282	0.397	0.585	156
TQ47	0.289	0.425	0.595	132
TQ48	0.261	0.389	0.599	119
TQ49	0.17	0.472	0.735	2
TQ50	0.272	0.406	0.599	116
TQ51	0.28	0.366	0.567	193
TQ52	0.312	0.407	0.566	196
TQ53	0.278	0.385	0.58	165
TQ54	0.258	0.39	0.602	110
TQ55	0.19	0.455	0.706	9
TQ56	0.269	0.393	0.594	137
TQ57	0.342	0.394	0.536	247
TQ58	0.32	0.385	0.546	229
TQ59	0.267	0.384	0.591	145
TQ60	0.287	0.411	0.588	148
TQ61	0.298	0.329	0.525	257
TQ62	0.304	0.375	0.552	218
TQ63	0.339	0.346	0.506	271
TQ64	0.273	0.405	0.598	125
TQ65	0.33	0.362	0.524	259
TQ66	0.306	0.413	0.574	180
TQ67	0.279	0.394	0.585	155
TQ68	0.297	0.397	0.571	183
TQ69	0.272	0.393	0.592	144
TQ70	0.21	0.418	0.665	21
TQ71	0.342	0.308	0.474	286
TQ72	0.296	0.374	0.558	207
TQ73	0.249	0.401	0.617	76
TQ74	0.254	0.4	0.612	88
TQ75	0.245	0.393	0.616	79
TQ76	0.323	0.405	0.556	210
TQ77	0.324	0.394	0.549	225
TQ78	0.344	0.351	0.504	273
TQ79	0.189	0.47	0.714	6
TQ80	0.29	0.418	0.591	146
TQ81	0.282	0.412	0.594	135
TQ82	0.274	0.376	0.578	173
TQ83	0.291	0.382	0.568	190
TQ84	0.308	0.37	0.546	232
TQ85	0.217	0.407	0.652	39

TQ86	0.235	0.394	0.626	64
TQ87	0.26	0.432	0.624	66
TQ88	0.265	0.411	0.608	100
TQ89	0.304	0.369	0.549	226
TQ90	0.268	0.422	0.611	90
TQ91	0.253	0.405	0.616	78
TQ92	0.304	0.362	0.543	239
TQ93	0.289	0.429	0.598	124
TQ94	0.31	0.391	0.558	208
TQ95	0.343	0.385	0.529	252
TQ96	0.316	0.376	0.544	238
TQ97	0.365	0.311	0.46	291
TQ98	0.319	0.393	0.552	220
TQ99	0.233	0.423	0.645	47
TQ100	0.337	0.348	0.508	267
TQ101	0.323	0.405	0.556	212
TQ102	0.362	0.319	0.468	288
TQ103	0.276	0.36	0.566	194
TQ104	0.259	0.402	0.609	96
TQ105	0.244	0.401	0.621	70
TQ106	0.285	0.372	0.566	195
TQ107	0.289	0.405	0.584	159
TQ108	0.221	0.427	0.659	26
TQ109	0.182	0.477	0.724	5
TQ110	0.258	0.42	0.619	71
TQ111	0.295	0.405	0.579	170
TQ112	0.295	0.406	0.579	169
TQ113	0.269	0.416	0.607	103
TQ114	0.257	0.403	0.61	93
TQ115	0.318	0.35	0.524	258
TQ116	0.39	0.23	0.371	302
TQ117	0.316	0.379	0.545	234
TQ118	0.29	0.373	0.563	199
TQ119	0.343	0.356	0.509	266
TQ120	0.316	0.387	0.55	223
TQ121	0.28	0.411	0.595	131
TQ122	0.166	0.47	0.739	1
TQ123	0.381	0.3	0.441	297
TQ124	0.372	0.31	0.454	293
TQ125	0.269	0.371	0.579	168
TQ126	0.329	0.352	0.517	264
TQ127	0.251	0.398	0.613	86
TQ128	0.261	0.386	0.597	126
TQ129	0.245	0.43	0.637	54

TQ130	0.318	0.396	0.555	215
TQ131	0.322	0.349	0.52	260
TQ132	0.275	0.416	0.602	111
TQ133	0.367	0.309	0.457	292
TQ134	0.289	0.398	0.579	167
TQ135	0.189	0.466	0.712	7
TQ136	0.3	0.375	0.556	213
TQ137	0.243	0.455	0.652	40
TQ138	0.229	0.44	0.657	30
TQ139	0.315	0.366	0.537	244
TQ140	0.42	0.282	0.402	301
TQ141	0.274	0.405	0.596	130
TQ142	0.323	0.393	0.548	227
TQ143	0.26	0.408	0.611	91
TQ144	0.314	0.398	0.559	203
TQ145	0.279	0.405	0.592	142
TQ146	0.315	0.359	0.533	250
TQ147	0.281	0.42	0.599	117
TQ148	0.306	0.393	0.562	201
TQ149	0.297	0.384	0.564	197
TQ150	0.362	0.311	0.462	290
TQ151	0.289	0.398	0.58	166
TQ152	0.329	0.396	0.546	231
TQ153	0.229	0.436	0.655	34
TQ154	0.314	0.361	0.535	248
TQ155	0.278	0.412	0.597	129
TQ156	0.252	0.368	0.594	138
TQ157	0.345	0.317	0.479	284
TQ158	0.35	0.33	0.485	281
TQ159	0.272	0.4	0.595	133
TQ160	0.246	0.383	0.609	95
TQ161	0.287	0.397	0.581	163
TQ162	0.253	0.408	0.617	75
TQ163	0.279	0.418	0.6	115
TQ164	0.377	0.311	0.452	295
TQ165	0.301	0.397	0.569	187
TQ166	0.348	0.348	0.5	274
TQ167	0.247	0.389	0.611	89
TQ168	0.267	0.398	0.598	121
TQ169	0.276	0.422	0.604	105
TQ170	0.369	0.342	0.481	283
TQ171	0.218	0.446	0.672	17
TQ172	0.309	0.405	0.567	192
TQ173	0.265	0.412	0.609	97

TQ174	0.19	0.465	0.71	8
TQ175	0.306	0.406	0.57	185
TQ176	0.244	0.401	0.622	69
TQ177	0.303	0.362	0.544	235
TQ178	0.395	0.296	0.429	300
TQ179	0.395	0.315	0.444	296
TQ180	0.27	0.395	0.594	136
TQ181	0.21	0.437	0.676	16
TQ182	0.267	0.406	0.603	109
TQ183	0.225	0.419	0.651	42
TQ184	0.285	0.39	0.578	172
TQ185	0.3	0.38	0.559	205
TQ186	0.216	0.424	0.662	22
TQ187	0.235	0.404	0.632	59
TQ188	0.221	0.422	0.656	32
TQ189	0.319	0.381	0.544	236
TQ190	0.311	0.383	0.552	219
TQ191	0.283	0.376	0.571	184
TQ192	0.327	0.334	0.505	272
TQ193	0.388	0.301	0.437	298
TQ194	0.362	0.329	0.476	285
TQ195	0.235	0.444	0.654	36
TQ196	0.234	0.445	0.655	33
TQ197	0.3	0.387	0.563	200
TQ198	0.29	0.382	0.569	188
TQ199	0.304	0.411	0.575	178
TQ200	0.215	0.413	0.658	27
TQ201	0.299	0.371	0.554	216
TQ202	0.299	0.403	0.574	181
TQ203	0.315	0.432	0.578	171
TQ204	0.239	0.414	0.635	57
TQ205	0.31	0.372	0.546	233
TQ206	0.233	0.438	0.653	38
TQ207	0.229	0.44	0.658	29
TQ208	0.229	0.425	0.65	44
TQ209	0.216	0.468	0.685	13
TQ210	0.309	0.372	0.546	230
TQ211	0.282	0.409	0.592	141
TQ212	0.234	0.41	0.637	55
TQ213	0.299	0.361	0.547	228
TQ214	0.238	0.378	0.613	87
TQ215	0.228	0.403	0.638	53
TQ216	0.21	0.462	0.687	12
TQ217	0.289	0.404	0.583	160

TQ218	0.266	0.402	0.602	112
TQ219	0.175	0.464	0.726	3
TQ220	0.245	0.383	0.61	94
TQ221	0.236	0.42	0.64	51
TQ222	0.241	0.421	0.636	56
TQ223	0.272	0.402	0.597	127
TQ224	0.257	0.387	0.601	114
TQ225	0.228	0.428	0.653	37
TQ226	0.256	0.376	0.595	134
TQ227	0.208	0.409	0.662	23
TQ228	0.306	0.41	0.573	182
TQ229	0.264	0.402	0.603	106
TQ230	0.19	0.447	0.702	10
TQ231	0.324	0.35	0.519	262
TQ232	0.294	0.373	0.559	202
TQ233	0.333	0.341	0.506	270
TQ234	0.314	0.363	0.536	246
TQ235	0.226	0.427	0.654	35
TQ236	0.287	0.391	0.577	176
TQ237	0.246	0.396	0.617	77
TQ238	0.247	0.414	0.627	63
TQ239	0.295	0.389	0.569	189
TQ240	0.264	0.421	0.615	82
TQ241	0.264	0.429	0.619	72
TQ242	0.314	0.368	0.54	241
TQ243	0.252	0.403	0.615	83
TQ244	0.29	0.374	0.563	198
TQ245	0.32	0.341	0.516	265
TQ246	0.32	0.345	0.519	261
TQ247	0.359	0.323	0.474	287
TQ248	0.381	0.291	0.433	299
TQ249	0.245	0.415	0.628	61
TQ250	0.26	0.377	0.592	143
TQ251	0.296	0.389	0.567	191
TQ252	0.223	0.431	0.659	24
TQ253	0.269	0.367	0.578	174
TQ254	0.24	0.399	0.624	68
TQ255	0.297	0.402	0.575	179
TQ256	0.283	0.427	0.601	113
TQ257	0.315	0.365	0.537	245
TQ258	0.345	0.382	0.525	256
TQ259	0.282	0.39	0.58	164
TQ260	0.309	0.387	0.556	211
TQ261	0.31	0.393	0.559	204

TQ262	0.28	0.417	0.599	120
TQ263	0.259	0.382	0.597	128
TQ264	0.345	0.395	0.534	249
TQ265	0.282	0.403	0.588	147
TQ266	0.328	0.391	0.544	237
TQ267	0.295	0.419	0.587	149
TQ268	0.29	0.402	0.581	162
TQ269	0.253	0.409	0.618	74
TQ270	0.257	0.374	0.593	140
TQ271	0.323	0.394	0.549	224
TQ272	0.256	0.416	0.618	73
TQ273	0.25	0.389	0.608	98
TQ274	0.365	0.36	0.497	277
TQ275	0.291	0.395	0.576	177
TQ276	0.203	0.431	0.679	14
TQ277	0.254	0.393	0.607	102
TQ278	0.316	0.371	0.539	242
TQ279	0.346	0.356	0.507	268
TQ280	0.34	0.366	0.519	263
TQ281	0.244	0.42	0.632	58
TQ282	0.178	0.47	0.725	4
TQ283	0.243	0.431	0.64	52
TQ284	0.292	0.387	0.57	186
TQ285	0.289	0.365	0.558	206
TQ286	0.329	0.366	0.527	255
TQ287	0.275	0.386	0.584	158
TQ288	0.225	0.403	0.642	49
TQ289	0.313	0.371	0.542	240
TQ290	0.261	0.39	0.599	118
TQ291	0.299	0.423	0.585	154
TQ292	0.255	0.396	0.608	101
TQ293	0.213	0.428	0.668	19
TQ294	0.263	0.37	0.584	157
TQ295	0.276	0.344	0.555	214
TQ296	0.271	0.415	0.605	104
TQ297	0.228	0.439	0.658	28
TQ298	0.27	0.429	0.614	85
TQ299	0.278	0.406	0.593	139
TQ300	0.327	0.407	0.554	217
TQ301	0.319	0.392	0.551	221
TQ302	0.245	0.417	0.63	60
